# The combination of all‐trans retinoic acid and Vorinostat protects neurons from oxidative stress via the Nrf2‐ARE pathway

**DOI:** 10.1002/alz70855_106950

**Published:** 2025-12-24

**Authors:** Chhanda Bose, Ashly Hindle, Adam Baker, Shane Smith, Sharda P Singh, J. Josh Lawrence

**Affiliations:** ^1^ Texas Tech University Health Sciences Center, Lubbock, TX, USA; ^2^ Garrison Institue on Aging, Lubbock, TX, USA; ^3^ TTUHSC, Lubbock, TX, USA; ^4^ Center for Translational Neuroscience and Therapeutics, Lubbock, TX, USA

## Abstract

**Background:**

A key feature of Alzheimer's disease (AD) is the accumulation of reactive oxygen species (ROS), which causes oxidative damage and mitochondrial dysfunction. Diet‐derived antioxidants such as all‐trans retinoic acid (ATRA) and oxidative stress (OS)‐responsive regulatory factors, such as nuclear factor erythroid 2‐related factor 2 (Nrf2), support exogenous and endogenous antioxidant defenses in neurons. Evidence suggests that ATRA and Nrf2 levels are decreased in the hippocampus of AD patients. Additionally, histone acetylation/deacetylation homeostasis is disrupted in AD. The present study seeks to determine whether the histone deacetylase inhibitor vorinostat and ATRA can synergistically or differentially regulate redox homeostasis to protect neuronal cells from OS

**Method:**

Mouse hippocampal neurons (HT22 cells) were pre‐treated with non‐cytotoxic doses of ATRA (5µM) and/or vorinostat (VOR; 0.5µM). After 24h, cells were challenged with 50µM H_2_O_2_ for 24h. Lipid peroxidation, mitochondrial oxidation, apoptosis, DNA fragmentation, Nrf2 expression, and antioxidant response enzymes were examined via colorimetric ELISAs, comet assay, TUNEL assay, flow cytometry, immunocytochemistry, and Western blots.

**Result:**

Pre‐treatment of HT22 cells with ATRA and VOR in combination was protective against 150 µM H_2_O_2_. 4‐HNE protein adducts, and apoptosis/necrosis were significantly attenuated by ATRA or VOR pretreatments (*p* <0.001). H_2_O_2_ showed numerous DNA strand breaks with an average of 94.8±4.7% of DNA in comet tails. However, pretreatments of ATRA and VOR significantly (*p* <0.001) decreased DNA breaks (9.4±0.3%‐17.1±0.61%). Pretreatments of either ATRA or VOR alone significantly increased Nrf2 protein levels (*p* <0.05) and led to increased downstream antioxidant enzymes HO‐1 and SOD. Although H_2_O_2_ alone induced Nrf2 protein expression, nuclear Nrf2 binding activity was significantly lower than in cells pre‐treated with ATRA (*p* <0.05, and *p* <0.001, respectively). Thus, nuclear Nrf2 activity does not result from generalized loss of total Nrf2 protein but may reflect impaired nuclear trafficking and transcription factor activity. Our observations suggest that the Nrf2 pathway is likely dysfunctional in hippocampal neurons after OS.

**Conclusion:**

It is evident that ATRA and VOR, alone or in combination, protect neuronal cells from OS via independent pathways. However, both pathways are important to initiate protection and could be useful to reduce OS during early disease pathogenesis and progression.

